# The *RhHB1*/*RhLOX4* module affects the dehydration tolerance of rose flowers (*Rosa hybrida*) by fine-tuning jasmonic acid levels

**DOI:** 10.1038/s41438-020-0299-z

**Published:** 2020-05-02

**Authors:** Youwei Fan, Jitao Liu, Jing Zou, Xiangyu Zhang, Liwei Jiang, Kun Liu, Peitao Lü, Junping Gao, Changqing Zhang

**Affiliations:** 1 Department of Ornamental Horticulture, College of Horticulture, China Agricultural University, Beijing, 100193 China; 2 Crop Research Institute, Guangdong Academy of Agricultural Sciences, Guangdong Provincial Key Laboratory of Crop Genetic Improvement, Guangzhou, Guangdong 510642 China

**Keywords:** Jasmonic acid, Abiotic

## Abstract

Phytohormones are key factors in plant responsiveness to abiotic and biotic stresses, and maintaining hormone homeostasis is critically important during stress responses. Cut rose (*Rosa hybrida*) flowers experience dehydration stress during postharvest handling, and jasmonic acid (JA) levels change as a result of this stress. However, how JA is involved in dehydration tolerance remains unclear. We investigated the functions of the JA- and dehydration-induced *RhHB1* gene, which encodes a homeodomain-leucine zipper I γ-clade transcription factor, in rose flowers. Silencing *RhHB1* decreased petal dehydration tolerance and resulted in a persistent increase in JA-Ile content and reduced dehydration tolerance. An elevated JA-Ile level had a detrimental effect on rose petal dehydration tolerance. RhHB1 was shown to lower the transient induction of JA-Ile accumulation in response to dehydration. In addition to transcriptomic data, we obtained evidence that RhHB1 suppresses the expression of the lipoxygenase 4 (*RhLOX4*) gene by directly binding to its promoter both in vivo and in vitro. We propose that increased JA-Ile levels weaken the capacity for osmotic adjustment in petal cells, resulting in reduced dehydration tolerance. In conclusion, a JA feedback loop mediated by an *RhHB1/RhLOX4* regulatory module provides dehydration tolerance by fine-tuning bioactive JA levels in dehydrated flowers.

## Introduction

Plants are challenged throughout their life cycle with different abiotic or biotic stresses, among which drought and dehydration are often the most limiting factors for crop production worldwide^1,2^. For the production and marketing of horticultural products, drought/dehydration also causes severe losses in quality and quantity^3,4^. Generally, water loss greater than 3–5% of most fresh horticultural products results in visual symptoms, such as shriveling and skin necrosis during postharvest handling^5^. To combat these types of stress, plants have evolved diverse and complex response strategies, which involve adaptations at the biochemical, physiological, cellular and molecular levels. Many of these are coordinated by the actions of phytohormones, which are crucial for both abiotic stress responses and the regulation of growth and development^6^. For example, the synthesis and signaling pathways of abscisic acid (ABA), ethylene (ET) and jasmonic acid (JA), etc., take part in regulating osmotic adjustment and other processes that confer drought tolerance^7,8^. Moreover, crosstalk between multiple pathways allows hormone homeostasis to be finely tuned, resulting in appropriate responses to the different stresses^9,10^.

JA and its methyl ester, methyl jasmonate (MeJA), are ubiquitous growth regulators naturally occurring in land plants^11,12^. Several enzymes have been identified to function in the JA biosynthesis pathway, including lipoxygenase (LOX), allene oxide synthase (AOS), allene oxide cyclase (AOC) and 12-oxophytodienoic acid reductase (OPR)^13^. In the JA signaling pathway, the binding of COI1 to JAZ is induced by the accumulation of JA, which results in the ubiquitination of JAZ by SCF^COI1^ and subsequent degradation by the 26S proteasome^14,15^. Numerous previous reports have suggested that JA plays an essential role in responses to tissue wounding caused by necrotrophic pathogens, insects, herbivores and mechanical stress^16^. JA is also recognized as a regulator of seed germination^17^, root growth^18^, and flower opening and senescence^19^. For example, numerous JA-related compounds have been found to accumulate in the flowers of *Petunia hybrida*, *Cymbidium faberi*, *Cattleya luteola*, etc.^20^. Exogenous jasmonates can promote tepal/petal senescence in *Dendrobium* and *Petunia*^21^ and can delay the senescence of nonclimacteric eggplant fruit^22^ and *Iris* flowers^23^.

Other studies have indicated that JA can play a direct and/or indirect role in abiotic stress responses^7^. For example, JA was observed to increase in *Carica papaya* seedlings^24^, *Pinus pinaster* plants^25^ and *Oryza sativa* (rice) leaves and roots^26^ when they were exposed to drought conditions. MeJA accelerates water loss but at the same time inhibits lipid peroxidation caused by dehydration in pear^27^, loquat^28^, and Romanesco wine grapes^29^. JA levels were also reported to transiently increase in water-stressed soybean (*Glycine max*) before declining to below wild-type levels^30^. Unstable elevated JA levels were also detected in drought-stressed roots of *Citrus paradisi* × *Poncirus trifoliata*^31^. In addition, JA signaling genes are responsive to abiotic stresses, such as cold and drought^26^, and *OsJAZ9* overexpression increased tolerance to salt and osmotic stress induced by mannitol^32^. The MeJA biosynthesis-related carboxyl methyltransferase gene was downregulated in response to dehydration in *Alstroemeria* cut flowers^33^. In oriental melon (*Cucumis melo* var. *makuwa* Makino), *CmLOX08* promoter fragments show responsiveness to ABA, SA, H_2_O_2_, and different abiotic stresses, e.g., salt and drought treatments^34^. These results indicate that JA biosynthesis and signaling show different patterns of responses and adaptations to different abiotic stresses. However, it is still unclear how plants fine-tune JA levels during stress responses.

Regulation of biological processes, including stress responses, often involves transcription factors (TFs). One of the TF families is the homeodomain-leucine zipper (HD-Zip) family, which is unique to plants and is involved in drought stress responses. HD-Zip proteins from this family are divided into four subfamilies (I-IV) based on the structural features of their amino acid sequences, which compose both a DNA-binding HD domain and a protein-protein interaction leucine Zip domain^35^. The γ-clade HD-Zip I subfamily comprises genes that function in adaptation to water deficit conditions^36,37^. For instance, *Arabidopsis thaliana AtHB7* and *AtHB12*, which are paralogs and encode proteins with highly similar sequences, are strongly and rapidly induced by water stress^38,39^. OsHOX22, belonging to the HD-Zip I subfamily in rice, improves drought tolerance by limiting ABA sensitivity^40^. Its homolog in sunflower (*Helianthus annuus*), HaHB4, provides increased survival rates under drought stress conditions when overexpressed in *A. thaliana*^41,42^. Although several previous reports have contributed to the functional understanding of HD-Zip I proteins under abiotic stress, it is still not well understood how γ-clade HD-Zip I TFs regulate the response to water deficit stress in different plant species.

Cut flowers of rose (*R*. *hybrida*) are the most widespread flowers in the ornamental horticulture industry worldwide; these flowers typically experience long-distance transport from production fields to consumers^43^. Dehydration tolerance is, therefore, critical for maintaining quality during postharvest handling. In previous studies, *RhNAC2* and *RhNAC3* were shown to improve dehydration tolerance by regulating cell wall- and osmotic adjustment-associated genes, respectively, in rose flowers^44,45^. Moreover, the regulatory module *RhABF2/RhFer1* contributes to the maintenance of Fe levels in rose petals to enhance dehydration tolerance^46^. Here, we investigated the role of RhHB1, a dehydration- and JA-induced HD-Zip I TF that is expressed in rose flowers. Our study provides new information regarding the function of RhHB1 in rose flowers during dehydration stress.

## Results

### Silencing of *RhHB1* decreases the dehydration tolerance of rose petals

**Fig. 1:**
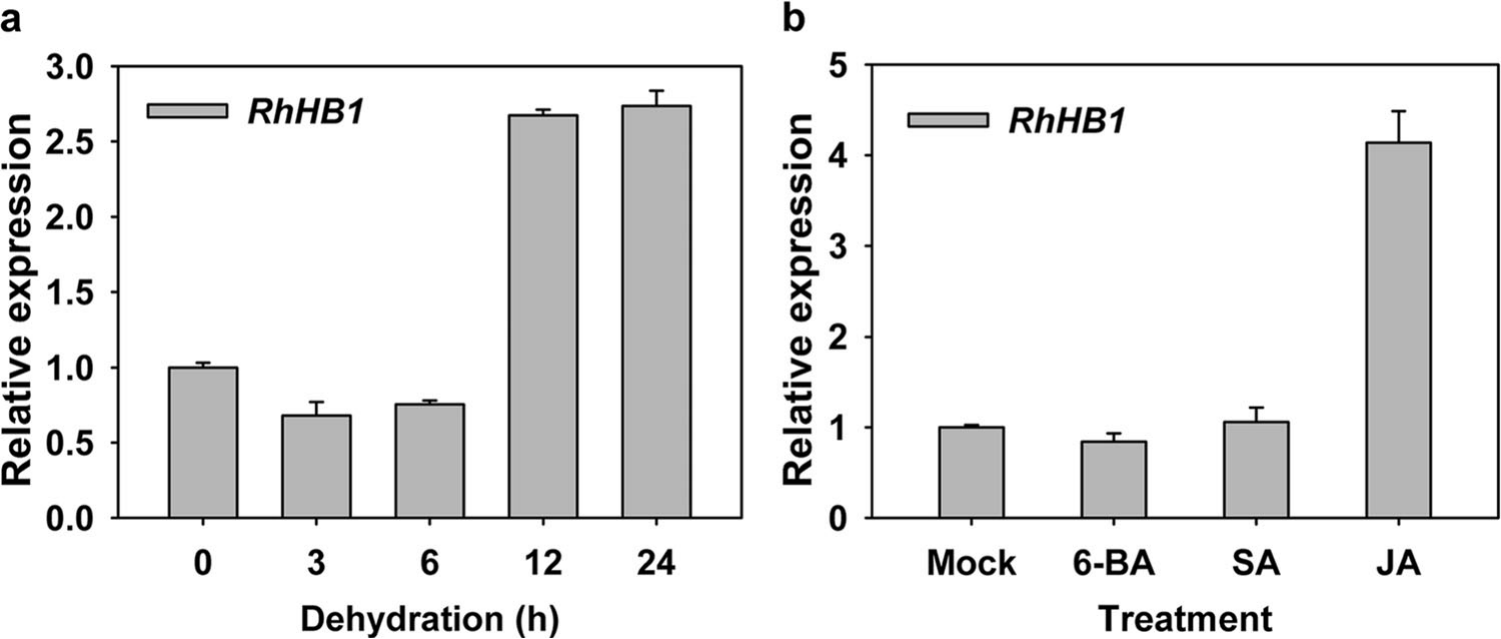
*RhHB1* expression in stressed rose petals. **a** *RhHB1* transcript levels in rose petals under dehydration. *RhUBI1* was tested as an internal control. **b** *RhHB1* expression levels after exogenous hormone treatments. 6-BA, 100 µM 6-benzylaminopurine; SA, 100 µM salicylic acid; JA, 100 µM methyl jasmonate. Rose flowers were treated at opening stage 2. All the values are the means ± SDs of five biological replicates

We previously reported that RhHB1, which belongs to the γ-clade HD-Zip I subfamily, mediates the antagonistic effect of GAs on ABA and ethylene action during rose petal senescence^47^. RhHB1 is also induced by both dehydration and ABA in rose petals^44^, so we investigated its possible role in dehydration tolerance. We first confirmed the expression patterns of *RhHB1* during a 24 h dehydration treatment or following exogenous hormone application by quantitative real-time PCR (RT-qPCR) experiments. The *RhHB1* expression level was relatively high under dehydration for 12 and 24 h (Fig. 1a), and we observed a fourfold accumulation of *RhHB1* transcripts at 24 h of JA treatment, while SA or cytokinin (CTK) applications did not alter *RhHB1* expression (Fig. 1b).

**Fig. 2:**
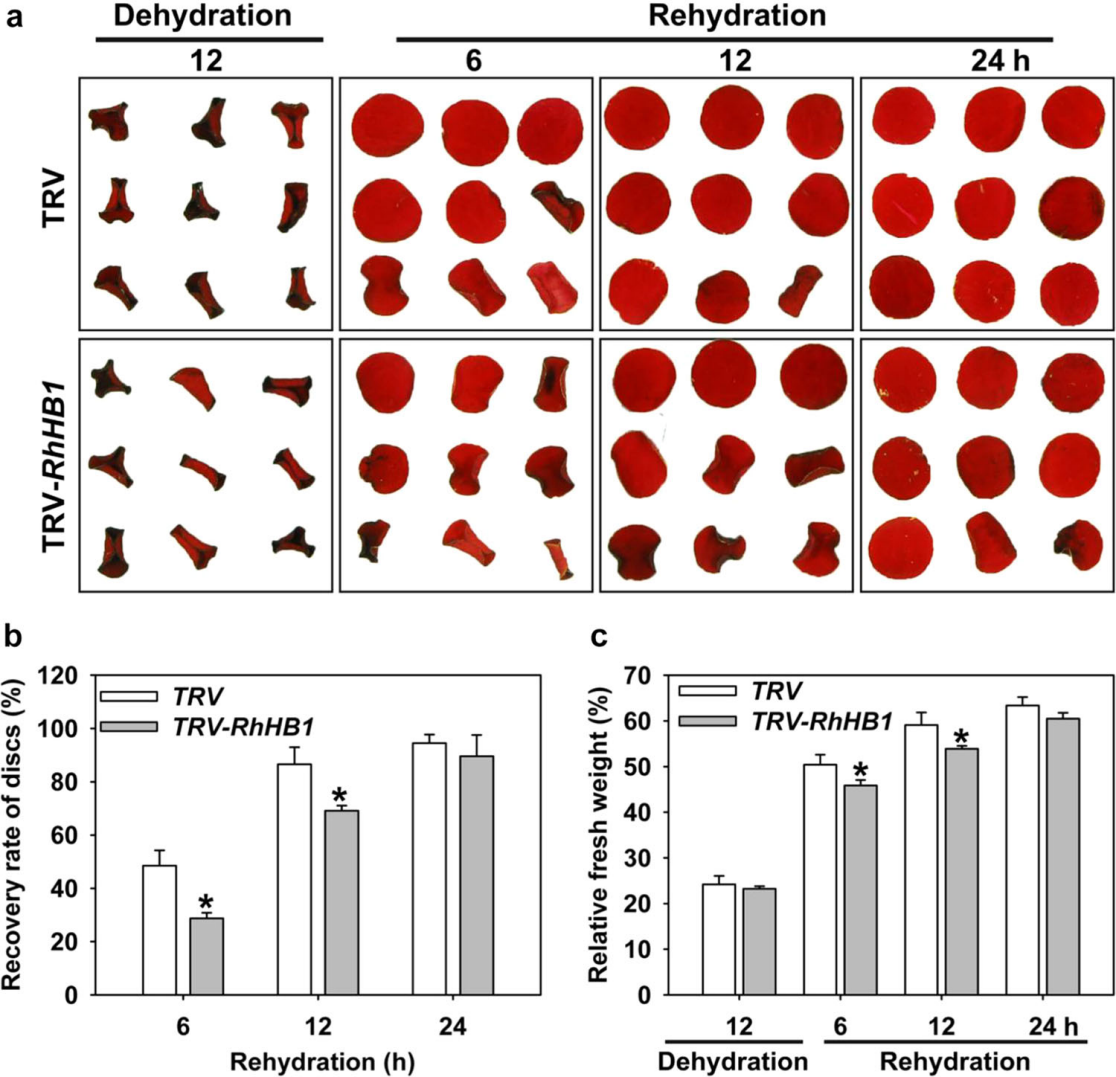
*RhHB1* silencing in rose petals by virus-induced gene silencing (VIGS). **a** Phenotype of *RhHB1*-silenced rose petals. Petals sampled from flowers at stage 2 were treated with VIGS. After infiltration, the discs were subjected to 12 h of dehydration followed by 24 h of rehydration in distilled water to observe recovery. The images were taken at the indicated intervals. **b** Recovery rate of *RhHB1*-silenced petal discs. Fully expanded petal discs were used (*n* = 5, *P < 0.05). **c** Relative fresh weight of *RhHB1*-silenced petal discs. The fresh weight was determined at the indicated time points (*n* = 5, *P < 0.05). All the values are the means ± SDs of five biological replicates, and the asterisks indicate significant differences according to Student’s *t*-test

We then silenced *RhHB1* in rose petals using virus-induced gene silencing (VIGS) and evaluated the dehydration tolerance as previously described^44^. Petal discs were infiltrated with *Agrobacterium tumefaciens* containing tobacco rattle virus (TRV)-*RhHB1* and dehydrated for 12 h before rehydration for 24 h (Fig. 2a). After 6 h of rehydration, the disc area had returned to 55% of the original value in the TRV controls compared with only 47% in the *RhHB1*-silenced discs, and this represented a significant difference (Fig. 2b). We also observed a smaller recovery of the fresh weight in silenced discs compared to the controls (Fig. 2c), indicating that *RhHB1* is involved in dehydration tolerance in petals.

### Global gene regulation by *RhHB1* in dehydrated rose petals

To further investigate the transcriptional regulation governed by RhHB1 and its role in dehydration tolerance, we performed an RNA-seq analysis of *RhHB1*-silenced and TRV control petals. Both the silenced and control petals were dehydrated for 12 h before RNA extraction. We identified 975 differentially expressed genes (DEGs) between the *RhHB1*-silenced and TRV control petals based on a *P*-value < 0.05 or min/max ≥2 with min >5, which eliminated genes expressed at a very low level. There were 537 upregulated DEGs and 438 downregulated DEGs (Data S1).

We conducted a plant-specific gene ontology (GO) analysis of these DEGs (http://www.geneontology.org/GO.slims.shtml). In the ‘biological process’ category, terms such as ‘response to abiotic stimulus’, ‘response to stimulus’ and ‘response to hormone’ were highly represented among the upregulated DEGs, while the ‘response to water’, ‘response to osmotic stress’, and ‘response to water deprivation’ terms were highly represented among the downregulated DEGs (Fig. S1). Notably, after further investigating the hierarchical clustering, we found that the more specialized child terms of ‘response of hormone’, including ‘responses to gibberellin’ and ‘responses to JA’, were enriched in the upregulated clusters (Data S2).

In our previous study, we showed that GAs are involved in delaying petal senescence primarily due to their antagonistic effect on ABA and ethylene action^44^. In the current study, we focused on DEGs enriched in ‘response to jasmonic acid’, which included 19 genes whose expression was upregulated in *RhHB1*-silenced petals compared to that in the TRV controls. These included four genes related to JA biosynthesis, such as *LOX*, *AOS*, and *AOC* genes, and 15 other genes that are responsive to JA, including TF-encoding genes (*MYB*, *WRKY*, and zinc finger family members) and some genes that encode functional proteins (Table 1). These results were consistent with RhHB1 playing a role in JA-related processes during dehydration. Expression profiles obtained through RNA-seq were validated by RT-qPCR analyses of four genes, all of which showed similar changes in expression in the RNA-seq and RT-qPCR data (Fig. S2).

**Table 1:** Differentially expressed genes involved in the biological process of the response to jasmonic acid in *RhHB*-silenced petals compared with TRV-transformed plants (petals infiltrated by empty vector)

ID	Description	TRV-*RhHB1*	TRV	Ratio	Adjusted *P*-value
*JA biosynthesis*					
RSA04711	Linoleate 13S-lipoxygenase 3-1	19.53	0.66	29.59	0.010465
RSA15517	Allene oxide cyclase, chloroplastic-like	12.67	3.8	3.33	0.017263
RSA15516	Allene oxide cyclase, chloroplastic-like	11.98	3.54	3.38	0.016298
RSA32325	Allene oxide synthase 1, chloroplastic	34.4	15.3	2.25	0.103875
*JA signaling*					
*Transcription factors*					
RSA21997	Transcription factor MYB44-like	15.26	2.46	6.2	1
RSA14486	Myb-related protein 308-like	37.59	7.59	4.95	3.05E-07
RSA21077	Protein REVEILLE 8-like	18.78	3.82	4.92	1
RSA52445	Myb-related protein 308-like	18.83	4.04	4.66	0.000252
RSA19906	Myb-related protein 305-like	481.12	159.01	3.03	0.000315
RSA34576	Probable WRKY transcription factor 46	19.75	7.15	2.76	0.743211
RSA28524	Zinc finger CCCH domain-containing protein 20	59.86	21.71	2.76	0.002431
RSA28522	Zinc finger CCCH domain-containing protein 20	58.14	21.63	2.69	0.003776
*Function proteins*					
RSA06085	Auxin-responsive protein IAA14-like	239.56	38.32	6.25	0.051357
RSA17592	Auxin-responsive protein IAA17-like	32.3	6.93	4.66	0.862039
RSA38142	Probable sodium/metabolite cotransporter BASS3	12.22	3.31	3.69	0.049198
RSA38141	Probable sodium/metabolite cotransporter BASS3	11.53	3.15	3.66	0.036746
RSA23848	Auxin-responsive protein IAA29	14.33	3.99	3.59	0.001150
RSA03246	Overexpressor of cationic peroxidase 3	6.78	3.04	2.23	1.000000
RSA20104	Ribulose bisphosphate carboxylase/oxygenase activase	46.67	17.02	2.74	0.778302

The ratio indicates the value of TRV-*RhHB1*/TRV

### *RhHB1* is required to optimize JA levels in dehydrated rose petals

**Fig. 3:**
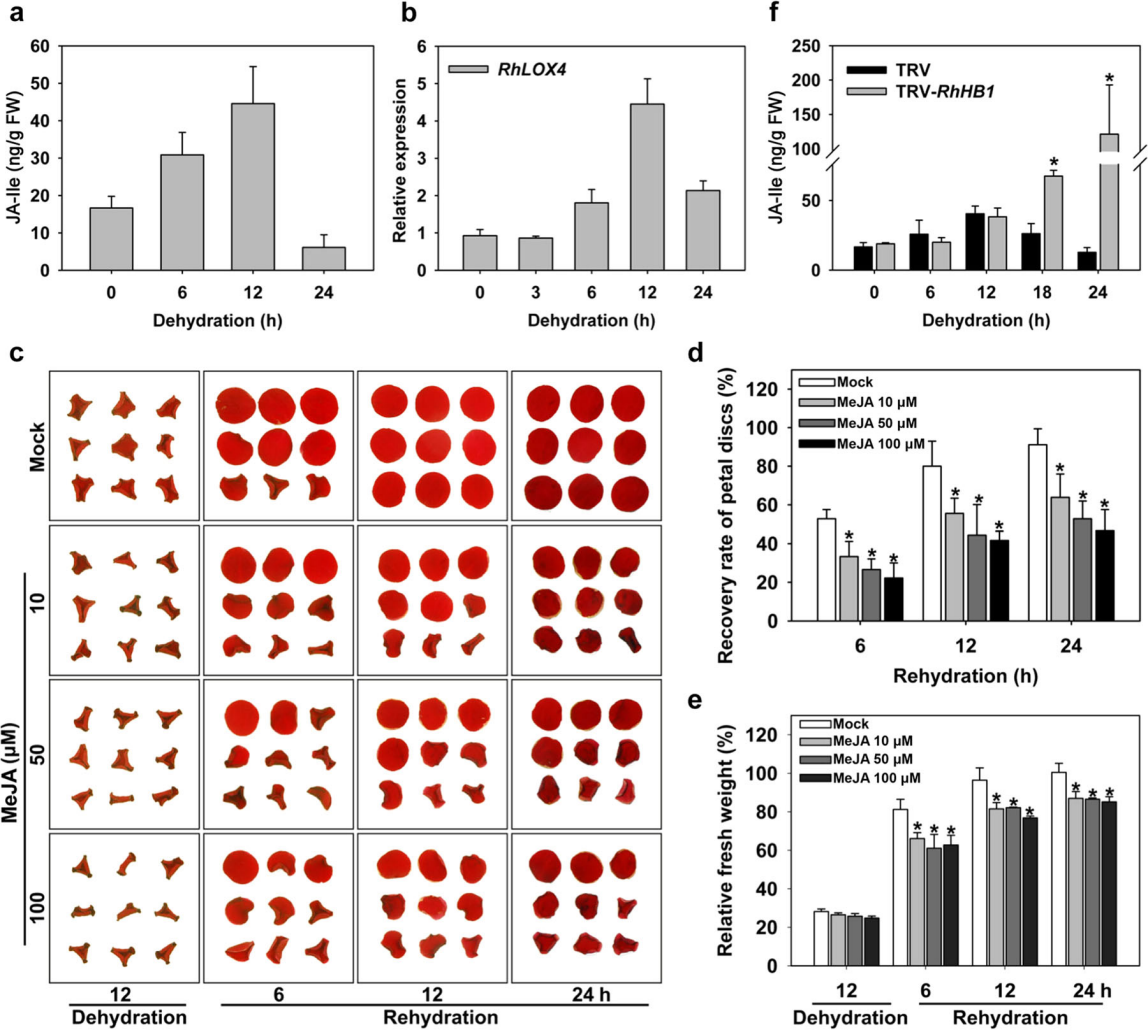
Effects of JA on rose petal dehydration tolerance. **a** JA-Ile levels in rose petals during 0–24 h of dehydration. FW, fresh weight. **b** *RhLOX4* expression in dehydrated rose petals. *RhUBI1* was used as an internal control (*n* = 5). **c** Phenotype of rose petals upon MeJA treatment. Rose petal discs were treated with different concentrations of MeJA (10, 50, and 100 µM) for 24 h. **d** Recovery rate of petal discs from MeJA-treated rose petals. Fully expanded petal discs were scored (*n* = 5, *P < 0.05). **e** Relative fresh weight of petal discs from MeJA-treated rose petals (*n* = 5, *P<;0.05). **f** Bioactive JA-Ile levels in TRV-infected control and TRV-*RhHB1* petals. *n* = 3, *P < 0.05. Rose flowers at opening stage 2 were treated, and all the values are the means ± SDs of biological replicates. The asterisks indicate significant differences according to Student’s *t*-test

To further investigate the function of JA in the dehydration tolerance of rose petals, we measured the levels of endogenous bioactive JA in dehydrated petals. We found that dehydration triggered an increase in JA-Ile levels in petals dehydrated for 12 h, while the levels were lower at the 24 h time point (Fig. 3a, b). This dynamic change in JA-Ile levels suggests the fine-tuning of JA metabolism in response to dehydration stress.

To test this hypothesis, we exogenously applied JA to rehydrating petal discs after a 12 h dehydration period to assess their recovery rates. As shown in Fig. 3c, after 6 h of rehydration, ~53% of the control disc areas recovered, which was significantly higher than the 33, 27, and 22% of the disc areas treated with 10, 50, and 100 µM JA, respectively. After 12 and 24 h of rehydration, a clear difference in recovery was observed between the control and JA-treated discs (Fig. 3d). Similarly, the recovered fresh weight of the control discs was higher than that of the JA-treated discs (Fig. 3e). These results suggest a detrimental effect of elevated JA levels on rose petal dehydration tolerance. We speculated that the reduced dehydration tolerance was related to altered JA levels in *RhHB1-*silenced petals. In addition, we found that, in contrast to the dynamic changes in the dehydrated control petals, the concentrations of JA continued to increase in the *RhHB1-*silenced petals at the 24 h time point (Fig. 3f), implying that silencing *RhHB1* alters the changes in JA concentration during dehydration. Taken together, these results suggest that dehydration-induced JA accumulation results in reduced petal dehydration tolerance and that RhHB1 is required to maintain optimal JA levels in dehydrated rose petals.

### RhHB1 directly represses the expression of lipoxygenase 4 gene during rose petal dehydration

**Fig. 4:**
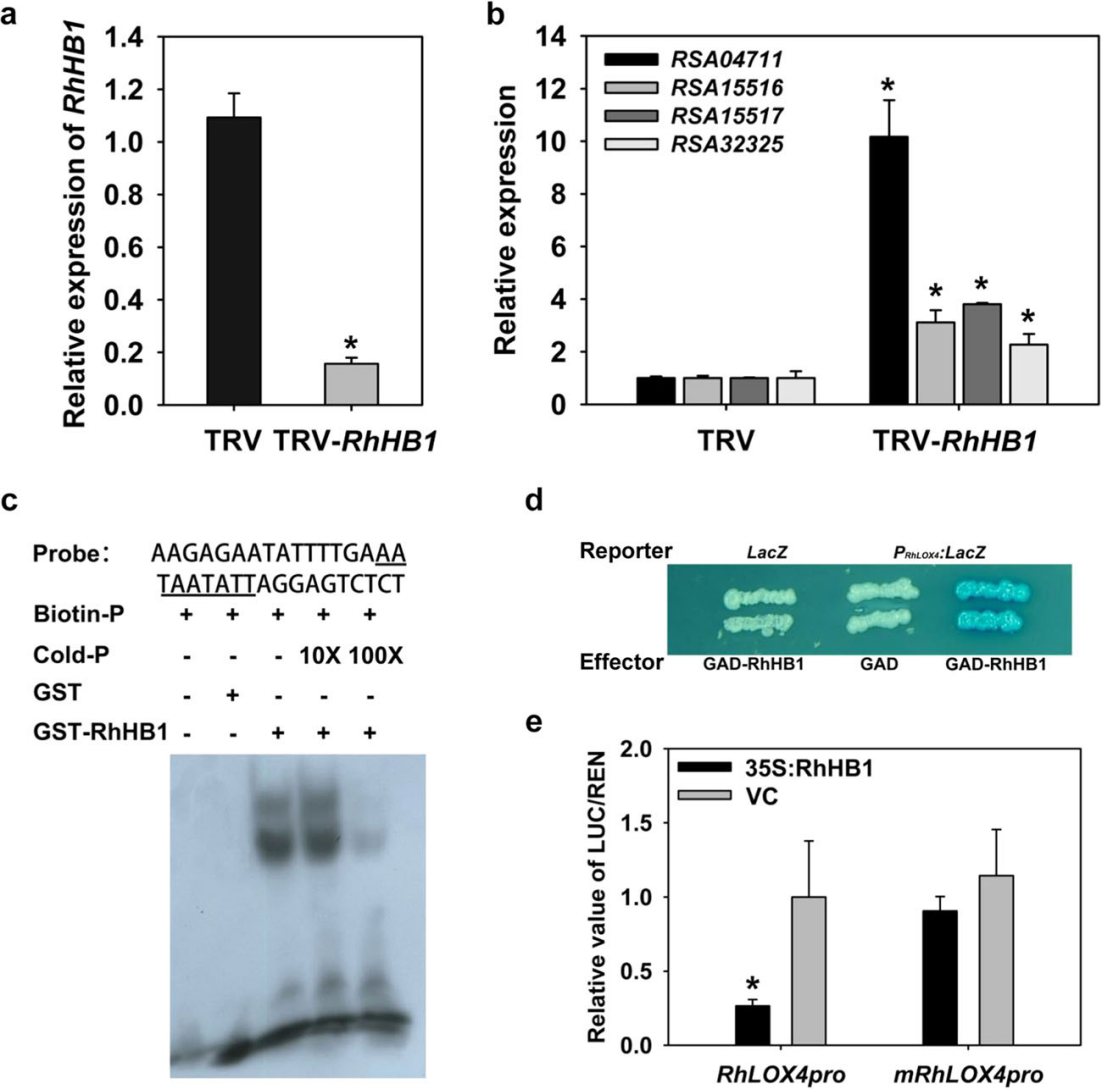
RhHB1 represses jasmonic acid (JA) biosynthesis by directly interacting with the *RhLOX4* promoter. **a** *RhHB1* expression levels in *RhHB1*-silenced rose petals as determined by RT-qPCR. The silenced petals were sampled and analyzed after 12 h of dehydration. **b** Expression levels of JA biosynthesis-related genes in *RhHB1*-silenced rose petals. **c** Gel-shift assay of RhHB1 binding to the *RhLOX4* promoter. The oligonucleotide fragment of −1270 to −1303 in the *RhLOX4* promoter was used as a probe containing the underlined core *cis*-element. Biotin-labeled probes (25 pM) were incubated with purified GST-RhHB1 protein (3µg), and competitors were added with nonlabeled probes at 10- and 100-fold concentrations. **d** Interaction between RhHB1 and the *RhLOX4* promoter in yeast. The vector combinations shown were introduced into yeast cells. The wild-type fragment (−1003 to −1396 bp) of the *RhLOX4* promoter was used in the *LacZ* reporter vector. **e** Transrepression activity of the *RhLOX4* promoter by RhHB1 in *N. benthamiana* leaves. The promoter region (0 to −1436 bp) of *RhLOX4* was used. mPro-*RhLOX4* is the same fragment as the mutated *cis*-element AcTcggAgc. The LUC/REN ratio was determined 3 days after leaf infiltration. All the values are the means ± SDs (*n* = 5 biological replicates, *P < 0.05), and the asterisks indicate significant differences according to Student’s *t*-test

We next investigated the potential regulatory links between *RhHB1* and genes related to JA biosynthesis. In the JA biosynthetic octadecanoic pathway, α-linolenic acid (18:3) is converted to cis(+)-oxophytodienoic acid through the successive actions of LOX, AOS, and AOC^13^. In our previous analysis, one *LOX* (RSA04711), one *AOS* (RSA32325) and two *AOC* (RSA15516 and RSA15517) genes were upregulated in *RhHB1*-silenced petals (Table 1). We confirmed the upregulation of these four genes in *RhHB1*-silenced petal samples by RT-qPCR. Consistent with the RNA-seq data, RSA04711 showed a more substantial increase in expression than did the other three genes (Fig. 4a, b). Using information derived from the recently released rose genome sequence^48,49^, we cloned the ~2000 bp upstream fragment of the predicted translational start site of each of the four genes and analyzed the *cis*-elements in their promoter regions. The predicted RhHB1 recognition sequence AATAATATT, which is highly similar to the reported *cis*-element involved in HD-Zip I TF recognition (CAAT(A/T)ATTG)^50^, was found only in the RSA04711 promoter region (−1280 ~ −1288 bp) (Fig. S3). Based on these results, we focused on the regulatory relationship between RhHB1 and RSA04711 in subsequent analyses.

We amplified the full-length sequence of RSA04711, which was predicted to have a 2328 bp ORF encoding a protein of 775 amino acids. Phylogenetic analysis revealed that RSA04711 is related to *AtLOX4* from *A. thaliana* (Fig. S4), so we named it *RhLOX4*. We further performed an electronic mobility shift assay (EMSA) to assess the interaction of RhHB1 and the *RhLOX4* promoter. A 34 bp fragment of the *RhLOX4* promoter, spanning positions -1270 to -1303 and containing the putative RhHB1 recognition sequence AATAATATT, was used as a probe. Compared with the GST control, only the GST-RhHB1 lane revealed DNA-binding signals when the labeled DNA probe was added (Fig. 4c). When we gradually increased the amounts of the unlabeled probe in the reaction mixture, the binding signals proportionally weakened (Fig. 4c). These data suggest that RhHB1 directly binds to the *RhLOX4* promoter region in vitro. We also performed a yeast one-hybrid assay to test the binding of RhHB1 to the *RhLOX4* promoter in vivo. The LacZ gene was driven by the *RhLOX4* promoter fragment (−1003 ~ −1,396) in the *P*_*RhLOX4*_*::LacZ* reporter construct. An effector construct (GAD-RhHB1) was generated to express the fusion protein comprising the RhHB1 ORF and the yeast GAL4 activation domain (GAD). We observed that GAD-RhHB1 but not GAD alone activated LacZ gene expression driven by the *RhLOX4* promoter fragment containing the RhHB1 recognition sequences (Fig. 4d).

We further conducted a dual-luciferase-based assay to test the activities of the *RhLOX4* promoter regulated by RhHB1 in *Nicotiana benthamiana* leaf cells. Effectors were constructed with the *LUCIFERASE* (*LUC*) gene driven by the *RhLOX4* promoter region (−1 ~ −1,396) (*Pro*_*RhLOX4*_*::LUC*) or a site-directed mutagenized version with a mutation within the *cis*-element AATAATATT (*mPro*_*RhLOX4*_*::LUC*). While the leaf cells were cotransformed with *35S::RhHB1* and *Pro*_*RhLOX4*_*::LUC* vectors, the relative LUC activities were lower than those in the cells cotransformed with *35S::RhHB1* and the *mPro*_*RhLOX4*_*::LUC* vector or in those cotransformed with the vector control and the effectors (Fig. 4e).

Taken together, the data suggest that during rose petal dehydration, RhHB1 directly represses *RhLOX4* expression, resulting in a decrease in JA biosynthesis.

### JA decreases dehydration tolerance by suppressing the expression of genes responsive to osmotic stresses

Petal dehydration tolerance of cut rose flowers is considered the ability of dehydrated flowers to fully open while rehydrating^44^. Generally, flower opening relies mostly on petal expansion, which is governed by changes in cell wall properties and cell turgor pressure^51^. To elucidate the molecular mechanisms underlying JA-induced petal dehydration tolerance, we reviewed the highly overrepresented GO biological processes related to petal expansion according to the RNA-seq data derived from *RhHB1*-silenced samples. Notably, only the term ‘response to osmotic stress’ (Go ID: 0006970) was overrepresented in the clusters representing downregulated DEGs (Fig. S1). The DEGs enriched in this term were annotated as being involved in the metabolism of osmolytes in petal cells, e.g., galactinol synthase, sugar transporter, metal ion transport, and solute carrier family 2 were identified (Table 2). We further checked the responses of these 24 DEGs to increased JA levels. RT-qPCR analysis confirmed that the expression levels of most of these DEGs were repressed when the rose petals were treated with exogenous JA (Table 2), which is consistent with JA restricting dehydration tolerance by limiting the capacity for osmotic adjustment of petal cells.

**Table 2:** Differentially expressed genes involved in the biological process of the response to osmotic stress in *RhHB1*-silenced and JA-treated petals

ID	Description	Ratio	Adjusted *P*-value	MeJA treatment	
				ECF	SD
RSA00921	Mitogen-activated protein kinase 3	0.52	0.036711	0.60	0.064805
RSA26383	3-Ketoacyl-CoA synthase 11-like	0.51	0.045716	0.64	0.090370
RSA13324	Sodium potassium root defective 3-like	0.47	0.047802	0.97	0.109413
RSA51312	Annexin-like protein RJ4	0.45	0.024355	0.62	0.098361
RSA34737	Fatty acyl-CoA reductase 1-like	0.44	0.014940	2.03	0.278192
RSA18163	Probable E3 ubiquitin-protein ligase XERICO	0.43	0.021571	0.98	0.187523
RSA49207	UDP-glycosyltransferase 74E1-like	0.42	0.022278	2.46	0.271695
RSA11779	Galactinol synthase 2	0.4	0.019530	2.73	0.302091
RSA39854	Sugar transporter ERD6-like 7	0.38	0.078520	0.62	0.018085
RSA29319	Sugar transporter ERD6-like 16	0.38	0.002830	0.59	0.105236
RSA34741	Fatty acyl-CoA reductase 1-like	0.36	0.006165	2.23	0.051153
RSA39855	Sugar transporter ERD6-like 7	0.36	0.000818	0.36	0.059674
RSA12559	UDP-glycosyltransferase 74E1-like	0.33	0.001462	3.65	0.797873
RSA42706	UDP-glycosyltransferase 74G1-like	0.33	0.000503	1.17	0.185479
RSA30845	Hydroquinone glucosyltransferase-like	0.32	0.398810	0.83	0.103464
RSA37707	Glutamate dehydrogenase 2	0.32	0.000132	0.80	0.065011
RSA14989	Zinc finger protein ZAT12-like	0.3	0.187691	0.12	0.066648
RSA42707	UDP-glycosyltransferase 74G1-like	0.29	0.002389	1.61	0.276270
RSA33855	Plasma membrane ATPase 1-like	0.28	0.000007	0.97	0.101301
RSA43028	MACPF domain-containing protein	0.27	0.001643	0.54	0.146397
RSA18939	Heavy metal-associated isoprenylated plant protein 24	0.13	0.000000	0.61	0.011454
RSA52357	9-*cis*-Epoxycarotenoid dioxygenase NCED6	0.04	0.293111	0.21	0.001865
RSA21074	Bidirectional sugar transporter N3	0.01	0.004232	0.26	0.114857
RSA49755	Peroxygenase 2-like	0.01	0.000338	1.26	0.227004

Ratio, the value of *RhHB*-silenced petals compared with that of TRV-infected plants. ECF, fold change in expression. The values are the means ± SDs (*n* = 5 biological replicates). MeJA treatment, flowers at stage 2 were treated with MeJA for 24 h

## Discussion

### JA plays a negative role in the dehydration tolerance of rose flowers

Dehydration and drought stress trigger changes in the levels of multiple hormones in plants^7,52^, and the associated hormone signaling networks enhance the tolerance or trigger programmed cell death in specific cells, tissues or organs, thereby allowing survival of adverse conditions^9^. JA and MeJA, which are products of fatty acid metabolism in plants, have multiple biological functions in responses to biotic and abiotic stresses^53^. Concentrations of endogenous JA have been proven to increase within a range of plant species, including *P. pinaster*^30^, *Cistus albidus*^54^, and *A. thaliana*^55^, when those plants are subjected to water deficit stress. In the current study, rose flowers showed an increase in endogenous JA levels in petals dehydrated for 12 h, while the levels were lower at the 24 h time point (Fig. 3a). A transient increase in JA concentration was also observed in water-stressed soybean^30^, desiccation-treated spear tips of *Asparagus officinalis*^56^ and drought-stressed citrus roots^31^. These results indicate that the roles of JA in response to water deficit stress are broadly conserved, albeit not well understood.

Exogenous application of JA has been reported to enhance tolerance to water deficit by inducing stomatal closure and thus decreasing transpiration via ABA-mediated mechanisms^57–59^. Previous reports have shown that increased JA concentrations enhanced the water transport capacity of the roots of *Phaseolus vulgaris*, *Solanum lycopersicum* and *A. thaliana*, thereby reducing tissue dehydration^60^. In rose petals, we observed that exogenous JA decreased dehydration tolerance by limiting petal expansion during subsequent rehydration (Fig. 3d–f). A similar result was reported, where MeJA delayed flower opening by inhibiting cell expansion in cut rose flowers^61^. Further analysis showed that relatively high JA levels in rose petals suppress the expression of genes closely involved in osmolyte metabolism and transport (Fig. S1 and Table 2). The genes involved in osmotic adjustment are critical to petal expansion in association with dehydration tolerance^44,62^. Similar results were also obtained where the application of JA and its isoleucine conjugate, JA-Ile, increased the detrimental effects of osmotic stress in *Vitis* cells^63^. The negative effects of JA in dehydration tolerance may be due to a stoma-independent regulatory mechanism in rose petals, which contrasts with the positive roles of JA-coordinated actions involving ABA and stomata in other species^57,59^.

### A feedback loop mediated by the *RhHB1/RhLOX4* module finely tunes JA-Ile concentrations during rose flower dehydration

The γ-clade members of HD-Zip I TFs are known to be regulated by different abiotic stress treatments^37^. For example, both *AtHB7* and *AtHB12* from *A. thaliana* show increased transcript levels responsive to water deficit or ABA treatment, allowing the fine-tuning of responses to mild water stress^39,63^. The expression of the homologs *OsHOX22* and *OsHOX24* is regulated by drought in rice^40,64^, and in the same clade, the expression levels of all three HD-Zip I transcription factor genes in wheat are induced under water deficit but show a substantial reduction following rehydration^37^. However, the functional mechanisms of these subgroup TFs in response to abiotic stresses such as water deficit have not been elucidated.

In our study, we measured the dehydration- and JA-induced γ-clade HD-Zip I TF *RhHB1* in rose flowers (Fig. 1)^47^ and observed that *RhHB1-*silenced petals showed poorer dehydration tolerance than did the controls (Fig. 2), likely due to altered responses to JA (Table 1 and Supplemental Data 2). We found that RhHB1 maintains optimal JA-Ile levels and provides dehydration tolerance in dehydrated rose petals (Fig. 3). The major steps of JA biosynthesis (*AOC1*/*4*, *OPR3*, and *LOX*) and signaling (*JAZ1*/*6*/*8* and *MYC2*) have been characterized^65^. Previous reports have shown that phytochrome-interacting factor 3 (PIF3) and WRKY62 suppressed the increase in expression levels of *LOX*s^66,67^; however, CITF1 and SPL7 upregulated the expression of *LOX3* and *LOX4* (ref. ^68^). Among the DEGs enriched in the response to jasmonic acid in rose petals, we screened 4 upregulated genes involved in JA biosynthesis (Table 1) and determined that RhHB1 binds directly to the *RhLOX4* promoter to suppress its expression both in vivo and in vitro (Fig. 4, Figs. S3 and S4).

**Fig. 5:**
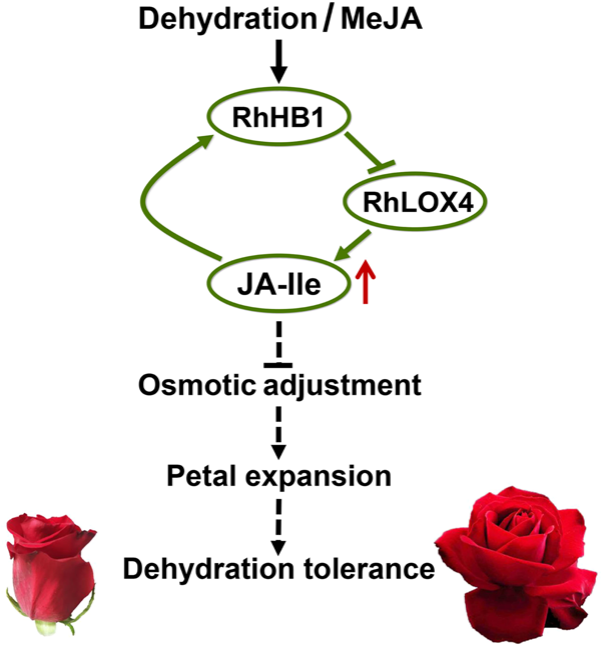
Model of the jasmonic acid (JA) feedback loop mediated by the *RhHB1/RhLOX4* regulatory module associated with dehydration tolerance in rose flowers. Dehydration-induced elevated JA-Ile levels inhibit rose petal expansion during rehydration by reducing the capacity for osmotic adjustment. Dehydration- and MeJA-induced *RhHB1* expression directly suppressed the expression of the JA biosynthesis gene *RhLOX4*, resulting in a decrease in JA-Ile accumulation. The red arrow represents increased JA-Ile levels during dehydration

Overall, we evaluated dehydration- and JA-induced *RhHB1* in rose flowers. Silencing *RhHB1* decreases dehydration tolerance by suppressing the expression of *RhLOX4*, a JA biosynthesis enzyme gene. Relatively high JA contents in rose flowers have detrimental effects on dehydration tolerance. RhHB1 suppression of *RhLOX4* expression results in decreased JA-Ile content during flower dehydration, causing negative feedback on dehydration-induced JA-Ile accumulation. The relatively high JA-Ile contents may weaken the osmotic adjustment ability, resulting in decreased tolerance of dehydrated rose flowers. We conclude that a feedback loop of JA mediated by the *RhHB1/RhLOX4* regulatory module provides tolerance by finely tuning the JA-Ile levels during dehydration of rose flowers (Fig. 5).

## Materials and methods

### Plant material and treatments

The rose flower opening index was defined as described by Ma et al.^69^ Rose (*R*. *hybrida* ‘Samantha’) flowers were used as materials in our study. The harvest and pretreatment procedures applied to the cut rose flowers were carried out according to the methods described by Jiang et al.^45^ The middle whorl petals were removed from rose flowers at opening stage 2 and were dehydrated by placing them horizontally in petri dishes for 3–24 h in a climate chamber with a temperature of 24 °C, a relative humidity of 40–-60%, and a constant light intensity of 120 µmol/m^2^/s. Each treated petal was weighed at 3, 6, 12, and 24 h during dehydration. The petal samples were then quickly frozen in liquid nitrogen and stored at −80 °C prior to RNA isolation.

For exogenous hormone treatments, rose stems at stage 2 were placed into a vase that contained 100 µM 6-benzylaminopurine (6-BA), 100 µM salicylic acid (SA) or 100 µM methyl jasmonate (MeJA) for 24 h. Mock samples were treated with DMSO without any phytohormones. A single stem was considered one biological replicate, and 5 biological replicates were sampled. Total RNA was extracted from the middle whorl petals of rose flowers using the hot borate method as previously described^70^.

### Cloning and plasmid construction

A cDNA template for gene cloning was synthesized with total RNA extracted from 12 h-dehydrated rose petals. The ORF of RhLOX4 was amplified with the primers RhLOX4F and RhLOX4R, which were developed according to the sequence of RSA04711 and were 3067 bp in length. For templates of gene promoter cloning, genomic DNA from rose leaves was extracted using the cetyltrimethylammonium ammonium bromide (CTAB) method^71^. Primers (RhLOX4pF1 and RhLOX4pR1) were designed on the basis of the ~2000 bp upstream sequence of the predicted translational start site of XM_024304926.1, which was annotated to LOX4 in the genomic database of *Rosa chinenses*^49^. The primer sequences used in this study are listed in Table S1.

The *RhHB1* VIGS vector was used as described by Lü et al.^34^ An *RhLOX4* VIGS vector (pTRV2*-RhLOX4*) containing a 5′ end 520-bp fragment of *RhLOX4* amplified by PCR with the primers RhLOX4F1 (with an *EcoR*I site at the 5′ end) and RhLOX4R1 (with an *Xho*I site at the 3′ end) was generated. The RhHB1 ORF was then ligated and fused to GST within a pGEX-2T vector to generate GST-RhHB1 proteins for an electrophoretic mobility shift assay (EMSA)^34^. GAD-RhHB1 and *P*_*RhLOX4*_*::LacZ* constructs containing the *RhHB1* ORF and *LOX4* promoter, respectively, were used in a yeast one-hybrid assay. For the dual-luciferase assay, *35S::RhHB1*, *Pro*_*RhLOX4*_*::LUC* and *mPro*_*RhLOX4*_*::LUC* constructs were used.

### RT-qPCR

All RT-qPCR analyses were conducted according to the methods of Lü et al.^34^ Briefly, RNA quality was confirmed by agarose gel electrophoresis. cDNA was synthesized from 1 μg of DNase-treated total RNA using a reverse transcription system (Promega, Madison, WI, USA) in a 20 μl reaction volume. A 2 μl cDNA aliquot was used as the template in a 20 µl RT-qPCR mixture using an Applied Biosystems StepOnePlus Real-Time PCR system (Applied Biosystems, Foster City, CA, USA) in standard mode with a Kapa SYBR^®^ Fast qPCR kit (Kapa Biosystems, Boston, MA, USA). A melting curve analysis was carried out for each reaction by slowly heating the sample from 60 to 95 °C at a rate of 0.3 °C/s, during which time the fluorescence was continuously measured. The quantity of target DNA was determined by the threshold cycle (C_T_), and relative gene expression values were calculated according to the 2^−ΔΔCT^ method, in which *RhUBI1* (GenBank accession JK622648) was used as an internal control^72^. All reactions were performed for five biological replicates. The RDML file for the RT-qPCR parameters is provided in the supplementary file (Data S3). All RT-qPCR primers used are listed in Table S1. Statistical analysis was conducted using Student’s *t*-test in SPSS version 17.0 (SPSS Inc., Chicago, IL, USA).

### Silencing *RhHB1* and *RhLOX4* in rose petals and petal discs using VIGS

VIGS of *RhHB1* and *RhLOX4* was conducted as described by Dai et al.^44^ In brief, *Agrobacterium tumefaciens* strain GV3101 was used to harbor pTRV1, pTRV2, pTRV2-*RhHB1*, and pTRV2-*RhLOX4* constructs. After 24 h of culture in Luria-Bertani (LB) media at 28 °C, the transformed *A. tumefaciens* cells were harvested after centrifugation at 5000 × *g* for 8 min and then resuspended in infiltration buffer with a final OD_600_ of ~1.8. A mixture of cultures containing an equal ratio (v/v) of pTRV1 and pTRV2, pTRV1 and pTRV2-*RhHB1*, or pTRV1 and pTRV2-*RhLOX4* were used in the VIGS experiments of the TRV-infected control, TRV-*RhHB1*, and TRV2-*RhLOX4* plants, respectively. Discs one-centimeter in diameter were collected with a hole punch from the rose flower petals at stage 2. After vacuum infiltration at 0.7 MPa in suspension buffer, the petals and discs were slightly rinsed and then maintained in deionized water at 8 °C for 3 d, after which they were subjected to an equilibration step at 23 °C for 1 d. The samples were subjected to 12 h of dehydration followed by 24 h of rehydration in distilled water. The fresh weight and recovery rate of the petal discs were determined at different time points, and the petals dehydrated for 12 h were collected to determine the degree of gene silencing using RT-qPCR.

### RNA-seq analysis, read processing, unigene assembly, and annotation

Total RNA samples were extracted from petals of *RhHB1*-silenced plants (TRV-*RhHB1*) and TRV-infected control plants using the hot borate method^70^. The RNA integrity and quantitation were confirmed using an Agilent 2100 Bioanalyzer (Agilent Technologies, Palo Alto, CA, USA). RNA sequencing libraries were generated as described by Zhong et al.^73^, and sequencing was performed on an Illumina HiSeq 2000 instrument. Low-quality reads, such as those containing adapters, barcode sequences and those with a *Q* value < 20 nucleotides, were filtered by a custom R script based on the ShortRead package^74^. The mapping of these reads was analyzed according to the GenBank virus (version 186) and rRNA databases^75^ using BWA^76^ with the default parameters, and the unmapped reads remained. De novo assembly of the unigenes was performed using Trinity with the strand-specific option ‘–SS lib type’ set to ‘F’ and ‘min kmer cov’ set to 2 (ref. ^77^). Redundant Trinity-generated unigenes were removed by further assembly of the reads using iAssemble^78^.

The resulting unigenes were compared to sequences in various protein databases by BLASTX^79^, including UniProt (Swiss-Prot and TrEMBL), GenBank nonredundant (nr) and *A. thaliana* protein databases (TAIR version 10), with a cutoff *E*-value of 1e^−5^. Gene annotation was performed based on the genome annotation database of *Rosa chinensis* ‘Old Blush’ (https://www.ncbi.nlm.nih.gov/genome/?term=rosa+chinensis)^49^. The annotated unigenes were subsequently mapped to GO terms in the UniProt database.

### JA-Ile extraction and quantification

JA-Ile was extracted and quantified as previously described^80^. Rose petals (50 mg) were sampled and ground into a powder in liquid nitrogen. Extraction was carried out by adding 0.5 mL of solvent (2-propanol:H_2_O:concentrated HCl [2:1:0.002, v/v/v]) and shaking at 100 rpm for 30 min at 4 °C, after which 1 mL of dichloromethane was then added, with shaking as done previously. After centrifugation at 13,000 × *g* for 5 min (4 °C), the lower phase was collected and concentrated under a stream of nitrogen before being redissolved in 0.1 mL of methanol. High-pressure liquid chromatography (HPLC) electrospray ionization tandem mass spectrometry was used to determine the JA-Ile content. Briefly, after redissolving in 0.1 mL of HPLC-grade methanol, the separation was achieved by elution via a linear gradient consisting of two phases: mobile phase A (distilled water with 0.1% formic acid) and mobile phase B (methanol with 0.1% formic acid). The gradient was as follows: *t* = 0 min, 30% B; *t* = 20 min, 100% B; and *t* = 25 min, 30% B. JA-Ile was measured at 322 nm. Jasmonoyl-isoleucine (OlChemim, Cat. #0146233) was added as an internal standard. Five biological replicates were used to conduct the statistical analysis by Student’s *t*-test via SPSS version 17.0.

### EMSAs and yeast one-hybrid assays

EMSAs and yeast one-hybrid assays were performed as previously described^47^. Briefly, *E. coli* BL21 cells were transformed with the RhHB1-containing pGEX-2T vector to express GST-RhHB1 fusion proteins and then incubated at 28 °C. Protein purification and EMSAs were performed according to the manufacturer’s instructions with glutathione sepharose 4B beads (GE Healthcare, Piscataway. NJ, USA) and EMSA kits (Pierce Biotechnology, Rockford, IL, USA), respectively. Biotin-labeled DNA fragments (RhLOX4-BF: Biotinol-5′- AAGAGAATATTTTGAAATAATATTAGGAGTCTCT-3′) were used as probes, while unlabeled DNA fragments were used as competitors.

For yeast one-hybrid assays, a GAD-RhHB1 fusion plasmid and a GAD control were cotransformed together with a *P*_*RhLOX4*_*::LacZ* construct into an EGY48 yeast strain as described in the Yeast Protocols Handbook (Clontech). The cotransformed yeast cells were subsequently cultured on SD/-Trp-Ura dropout plates containing X-gal to observe the color development of the yeast colonies.

### Dual-luciferase assays in tobacco leaves

The RhHB1 ORF was amplified and then cloned into a pGreenII 0029 62-SK vector^81^ to generate an effector construct (*35S::RhHB1*). The region (−1 ~ −1396) and site-directed mutagenized sequences of the *RhLOX4* promoter were ligated a pGreenII 0800-LUC double-reporter vector^81^ as effectors (*Pro*_*RhLOX4*_*::LUC*; m*Pro*_*RhLOX4*_*::LUC*). A pGreenII 0800-LUC vector harboring *CaMV35S::REN* served as a positive control. The LUC/REN ratio represented the repression of the *RhLOX4* promoter by RhHB1. All constructs were transformed into *A. tumefaciens* EHA105. The transformed bacteria were then cultured for 12 h before harvesting for infiltration into *N. benthamiana* leaves. At three days after infiltration with a mixture of TF and promoter cultures (1:5), the LUC and REN activity in the tobacco leaves was assayed. Five biological replicates were subjected to statistical analysis by Student’s *t*-test via SPSS version 17.0.

## Supplementary information

Figure S1-S5

Table S1

Data S1

Data S2

Data S3
