# Supplementary material for: The RhHB1/RhLOX4 module affects the dehydration tolerance of rose flowers (Rosa hybrida) by fine-tuning jasmonic acid levels
Source: Hortic Res. 2020 May 2;7:74. doi: 10.1038/s41438-020-0299-z (PMC7195446; doi:10.1038/s41438-020-0299-z)
Supplement: Supplementary file 1 — Figure S1-S5 [file 41438_2020_299_MOESM1_ESM.doc]

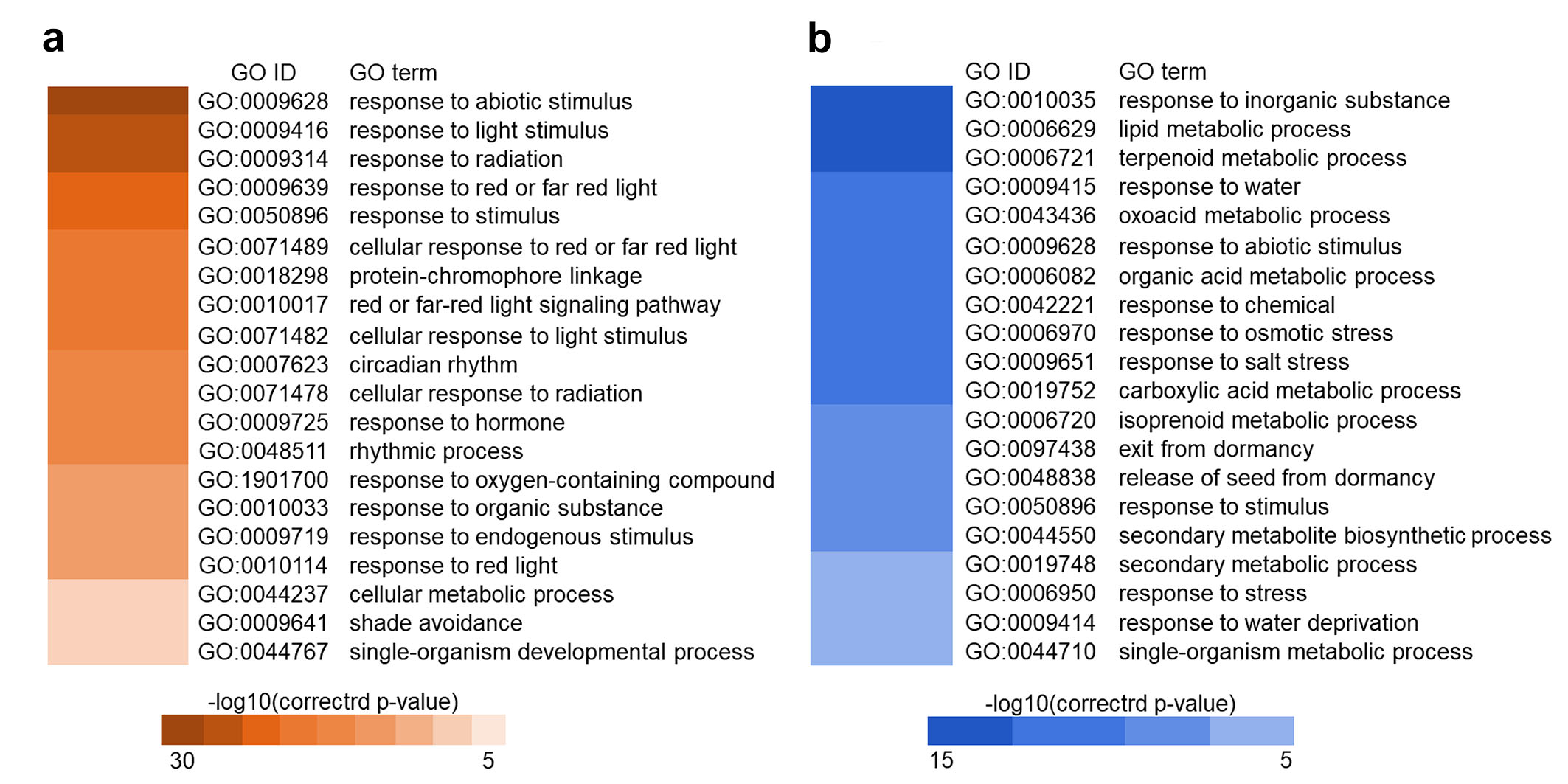


**Figure S1 GO terms analysis of DTGs in *RhHB1*-silenced rose petals.**

Gene Ontology (GO) analysis of differentially expressed genes in *RhHB1*-silenced rose petals compared with those in the TRV controls.

(a) GO term in the biological process analysis of upregulated genes.

(b) GO term in the biological process analysis of downregulated genes.

Top 20 GO categories are shown with color codes representing the -log10 (P-value).


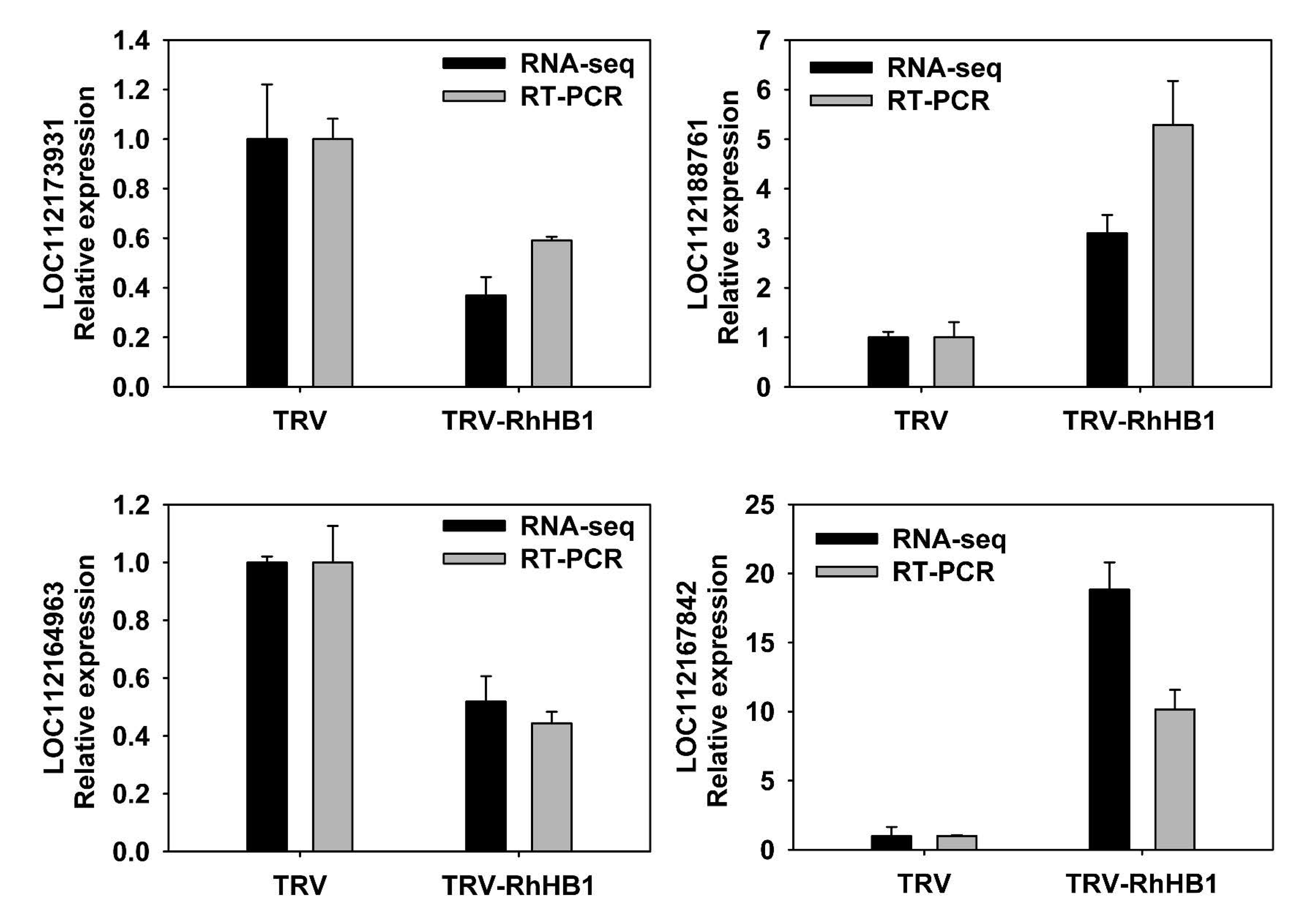


**Figure S2 Verification of RNA-seq results by qRT-PCR.**

qRT-PCR analysis of selected genes. *RhUBI1* was used as an internal control. Values are means ± SD (n=3).


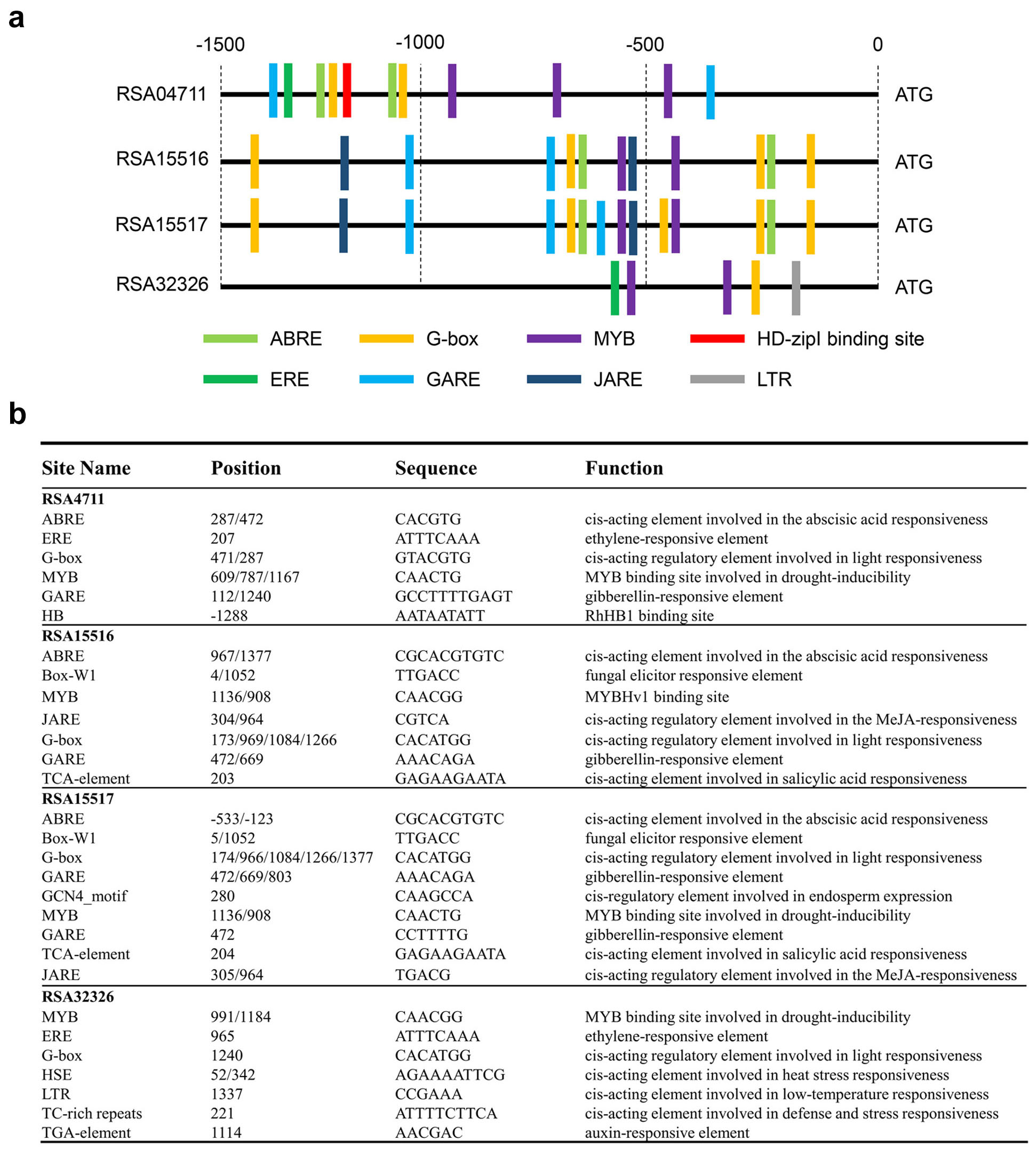


**Figure S3 Promoter analysis of JA biosynthesis genes.**

Analysis of potential cis-elements in the lipoxygenase genes promoter region. The position and putative sequences of cis-acting elements are listed. The predicted cis-acting elements were obtained using the PLACE program.


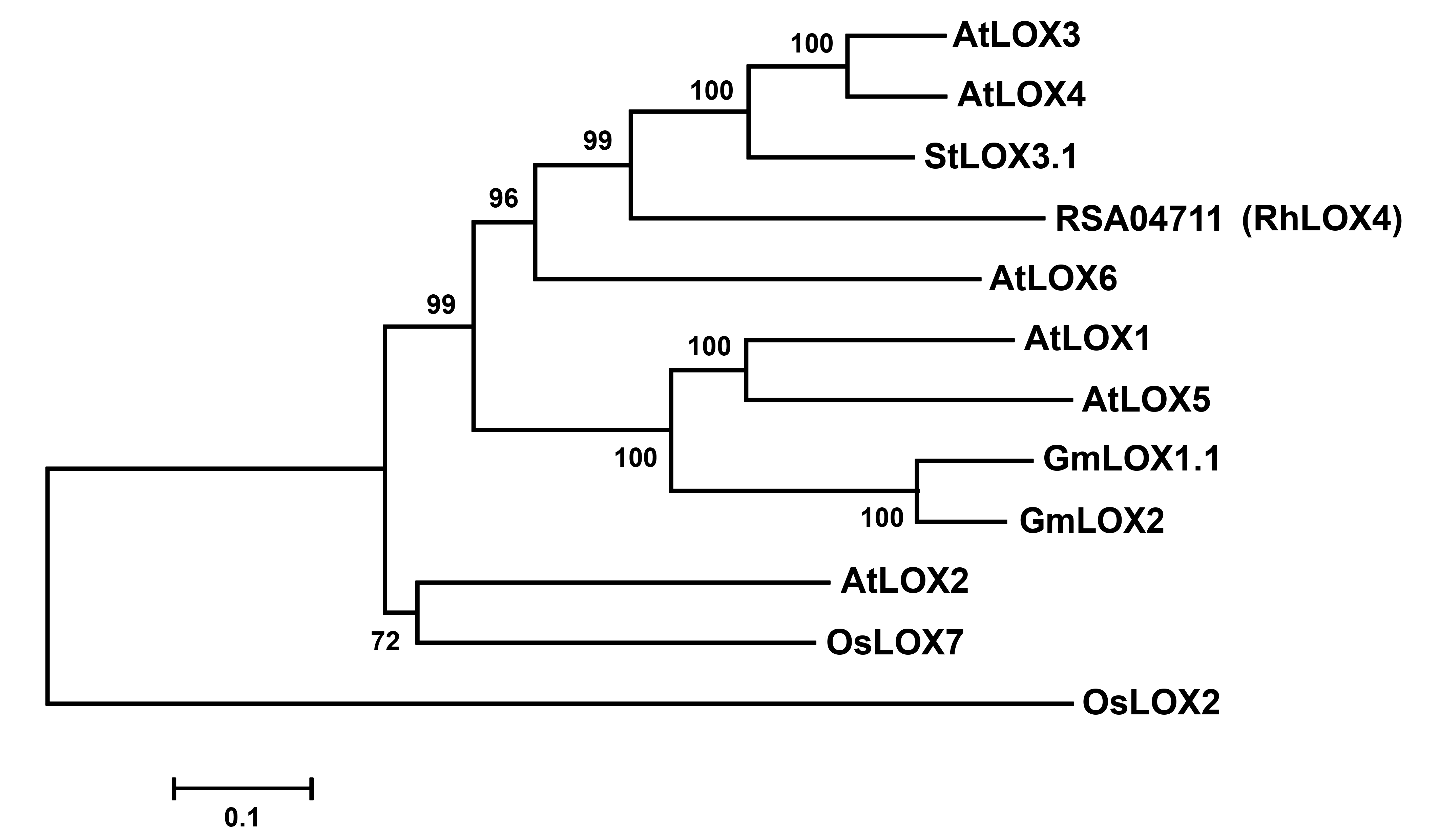


**Figure S4** **Phylogenetic analysis of RhLOX4.**

Phylogenetic tree generated using various Lipoxygenase protein sequences. AtLOX1 (AT1G55020), AtLOX2 (AT3G45140), AtLOX3 (AT1G17420), AtLOX4 (AT1G72520), AtLOX5 (AT3G22400), AtLOX6 (AT1G67560) from *Arabidopsis thaliana*, GmLOX1.1 (NP_001236153.2), GmLOX2 (NP_001237685.2) from *Glycine max*, OsLOX7 (XP_015650717.1), OsLOX2 (XP_015620638.1) from *Oryza sativa*, StLOX3.1 (NP_001275115.1) from *Solanum tuberosum* and RhLOX4 (LOC112167842). Bootstrap values indicate the divergence of each branch, and the scale indicates branch length. The percentages of bootstrap support are shown on the branches. These values were calculated from 1000 bootstrap re-samplings.


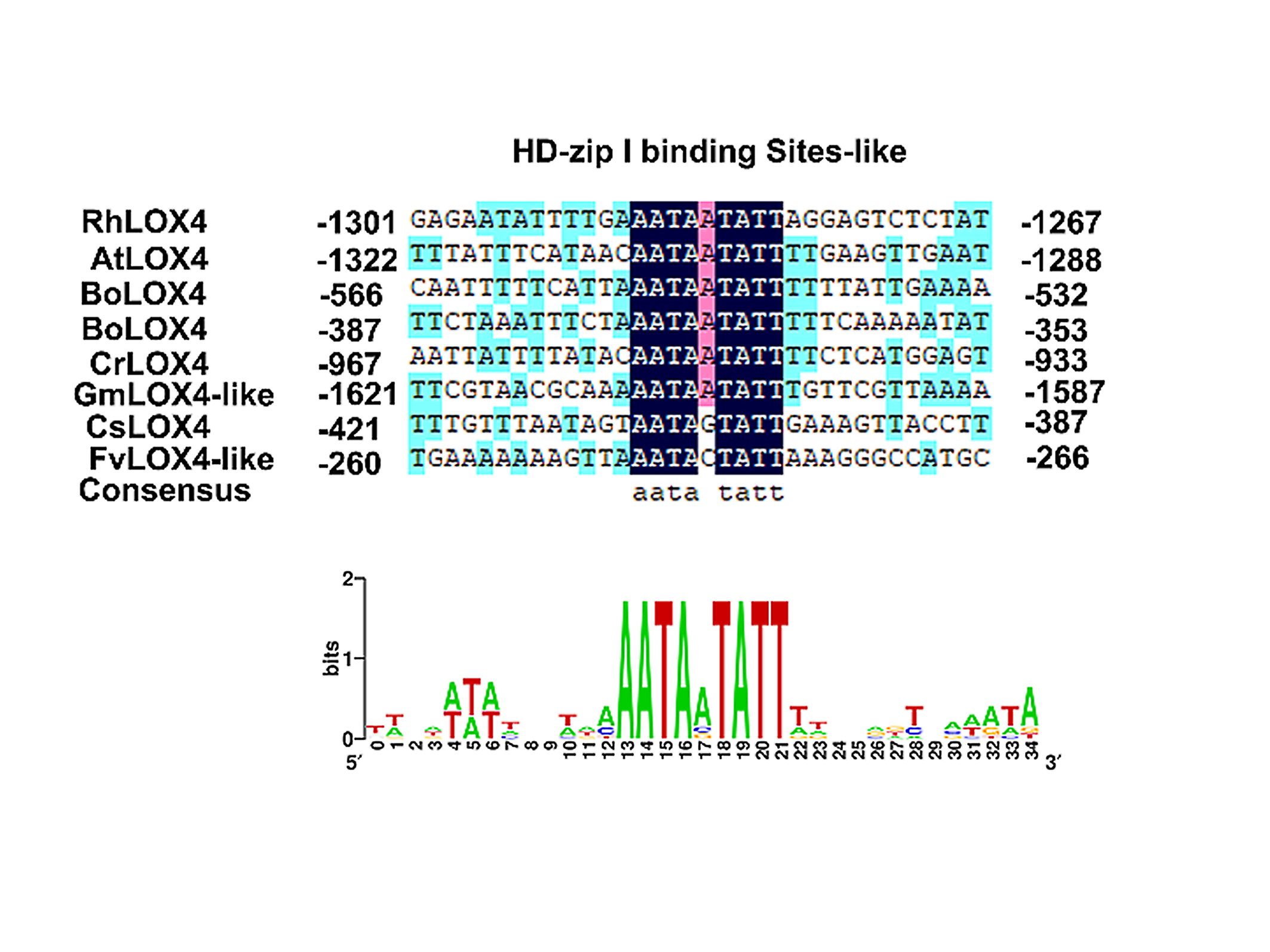


**Figure S5 Position and conservation of the HD-Zip I binding sites in the promoter of *LOX* genes in different plant species.**

The HD-Zip I binding motifs (AATANTATT) were found in the LOX promoters from various plant species. AtLOX4 (AT1G72520 from Arabidopsis, GmLOX4-like (LOC100810270) from *Glycine max*, BoLOX4 (LOC106327768) from *Brassica oleracea*, CrLOX4 (LOC17896557) from *Capsella rubella*, and CsLOX4 (LOC104712857) from *Camelina sativa*, FvLOX4-like (MG708227.1) from *Fragaria vesca* and RhLOX4 (LOC112167842). The sequence was obtained from genomic sequences. Sequence analysis was processed by DNAMAN software and online tools ([http://weblogo.berkeley.edu](http://weblogo.berkeley.edu/)).
