# Supplementary material for: The RhHB1/RhLOX4 module affects the dehydration tolerance of rose flowers (Rosa hybrida) by fine-tuning jasmonic acid levels
Source: Hortic Res. 2020 May 2;7:74. doi: 10.1038/s41438-020-0299-z (PMC7195446; doi:10.1038/s41438-020-0299-z)
Supplement: Supplementary file 2 — Table S1 [file 41438_2020_299_MOESM2_ESM.docx]

**Table S** Primer sequences used in this study.

| Primer name |  | Sequence(5’-3’) |
| --- | --- | --- |
| **For isolation of RhHB1 in rose** | | |
| RhHB1-F | Forward | ATGTTAGAGGTAGACCGAGTT |
| RhHB1-R | Reverse | TCAAGACCAAAAATCCCACC |
| **For isolation of RhLOX4 in rose** | | |
| RhLOX4F | Forward | ATGAAGCCAAAGCTGAGTGA |
| RhLOX4R | Reverse | TCAAACTGATATACTATTAGG |
| **For Promoter amplification** | | |
| RhLOX4pF1 | Forward | TTAATCATGATACCCTAGAACTAT |
| RhLOX4pR1 | Reverse | TTCCTCTGCATTTGTTTAAA |
| **For vector constructions of yeast one hybrid** | | |
| RhLOX4-Y1H-F | Forward | GCTGAATTCATGAGAGTGTACATGATACAA |
| RhLOX4-Y1H-R | Reverse | GCACTCGAGATAACTCCAGCGTATCATGTTTA |
| RhHB1-Y1H-F | Forward | GCCATGGAGGCCAGTGAATTCATGTTAGAGGTAGAC |
| RhHB1-Y1H-R | Reverse | ACGATTCATCTGCAGCTCGAGTCAAGACCAAAAATC |
| **For vector constructions of transcription activation in tobacco leaves** | | |
| RhHB1-EcoRI-F: | Forward | CAGAATTCATGTTAGAGGTAGACCGAGTT |
| RhHB1-XhoI-R: | Reverse | CGACTCGAGTCAAGACCAAAAATCCCACC |
| RhLOX4-Xho1-F: | Forward | GCACTCGAGATGAGAGTGTACATGATACAA |
| RhLOX4-SalI-R: | Reverse | CGTCGACTTCCTCTGCATTTGTTTAAA |
| RhLOX4-mut-F： | Forward | AAGAGAATATTTTGAACTCGGAGCAGGAGTCTCTATA |
| RhLOX4-mut-R： | Reverse | TATAGAGACTCCTGCTCCGAGTTCAAAATATTCTCTT |
| **For vectors construction VIGS of in rose petals** | | |
| RhLOX4F1 | Forward | GGGGAAAGAAAAGACCTGTCCA |
| RhLOX4R1 | Reverse | CAGTTGCCGAGCTATAGAGTTCA |
| RhLOX4-VIGS-F | Forward | GGAATTCGGGGAAAGAAAAGACCTGTCCA |
| RhLOX4-VIGS-R | Reverse | TCACTCGAGCAGTTGCCGAGCTATAGAGTTCA |
| RhHB1-VIGS-F | Forward | CCGGATCCAGAGGAAAGGAACAATAGCAT |
| RhHB1-VIGS-R | Reverse | CGACTCGAGGAACATTTTTCTTATTTTTACAC |
| **For vectors construction EMSA** | | |
| RhLOX4-EMSA-F | Forward | AAGAGAATATTTTGAAATAATATTAGGAGTCTCT |
| RhLOX4-EMSA-R | Reverse | AGAGACTCCTAATATTATTTCAAAATATTCTCTT |
| RhHB1-EMSA-F | Forward | GCGGATCCATGTTAGAGGTAGACCGAGTT |
| RhHB1-EMSA-R | Reverse | GCGAATTCTCAAGACCAAAAATCCCACC |
| **For qRT-PCR analysis in rose** | | |
| RSA12559-qF: | Forward | GTTTTAGATGTGGCACGGCG |
| RSA12559-qR: | Reverse | ACATCGGAGAGAAACGTCGG |
| RSA39855-qF: | Forward | TCCCGAGTCTCCAAGATGGT |
| RSA39855-qR: | Reverse | CCGCGAAGTTTCTGTAGTGC |
| RSA39854-qF: | Forward | TTCCCGAGTCTCCAAGATGG |
| RSA39854-qR: | Reverse | AGCATCTTTCCCGCGAAGT |
| RSA00921-qF: | Forward | GGCGGCCAGTACATTCAGTA |
| RSA00921-qR: | Reverse | TCTTCATCGCCACCATCTCG |
| RSA33855-qF: | Forward | TACCTTCTTCGGCAAGGCTG |
| RSA33855-qR: | Reverse | CGACCATTCCCACAGCGATA |
| RSA21074-qF: | Forward | AAGACGTGGACGGCAAAGAT |
| RSA21074-qR: | Reverse | GAAGAAGCTACTCTCGCGCT |
| RSA26383-qF: | Forward | CATCTTACTCGTCCCGCTCC |
| RSA26383-qR: | Reverse | GAACCAAGTCCTCGACGGTT |
| RSA51312-qF: | Forward | GAGCGGTGTACCGTTGGATA |
| RSA51312-qR: | Reverse | AGTGCTTGTAGCGAAGCTGG |
| RSA49755-qF: | Forward | GCTGGAATGGGGACTTCTGT |
| RSA49755-qR: | Reverse | GCTTCCATCAAAGCAGCGTC |
| RSA34741-qF: | Forward | CAATTTGCCTCTTGTGGTCG |
| RSA34741-qR: | Reverse | AAGCATGTCAGCTTGCCTTTG |
| RSA11779-qF: | Forward | TACTTCAACGCCGGCATGTT |
| RSA11779-qR: | Reverse | ACAAGGTTGTACACCGGAGG |
| RSA37707-qF: | Forward | GGTACATGACTCGGGCCTTC |
| RSA37707-qR: | Reverse | GCTACCCGGTTTACACCCAA |
| RSA18939-qF: | Forward | AAGAAGGTCCTCTCCGGTGT |
| RSA18939-qR: | Reverse | CGTAGGCTCCGGCAACATAA |
| RSA43028-qF: | Forward | ATCTTCGGGCATAACCTCGG |
| RSA43028-qR: | Reverse | TCAAAAGGCCAGACGACGAC |
| RSA18163-qF: | Forward | GGTCTGTCGAACTTTCCGGG |
| RSA18163-qR: | Reverse | GGCGCCACTCATTACTTGGA |
| RSA30845-qF: | Forward | GCCATGGCTTTGTCGTTGTT |
| RSA30845-qR: | Reverse | CACCCATTTGTAGGCCTCGT |
| RSA14989-qF: | Forward | GACAGCCAGTCGTCTTCTCC |
| RSA14989-qR: | Reverse | TAGCAAACTCCAACCCGCAT |
| RSA49207-qF: | Forward | TGGGTAGTCCGAGAAACGGA |
| RSA49207-qR: | Reverse | CTGCCTTGTGAGCCAAGACT |
| RSA13324-qF: | Forward | TCTAACCCTAGACTGCGCCT |
| RSA13324-qR: | Reverse | AACCTGGTTACGAGAGCGTG |
| RSA34737-qF: | Forward | CGGCTTCCTTGGAATGGTGT |
| RSA34737-qR: | Reverse | CTTGCATGCGATGTGTTGCT |
| RSA29319-qF: | Forward | TGCGGACTTTCATGGTCGAA |
| RSA29319-qR: | Reverse | GGAGATTTTTGGGCGCGATT |
| RSA47206-qF: | Forward | ACAACCCGGTCCGAACTTTT |
| RSA47206-qR: | Reverse | TTGTCGAAGAACAGCCGGAA |
| RSA47207-qF: | Forward | TTGTGTCTCTGGGTGGTAACAA |
| RSA47207-qR: | Reverse | AAAACTCCGCCCTTCCGATG |
| RSA52357-qF: | Forward | TGGTCTACTTCAACGGACGC |
| RSA52357-qR: | Reverse | CCGTCCGATCGTTTCGAGAT |
| RhLOX4-qF: | Forward | TGCATATCGAGACTGGTGGC |
| RhLOX4-qR: | Reverse | TGAGGAGTCCATCTGTTGCG |
| RSA15516-qF: | Forward | TGACTTACGAGGACACGAGC |
| RSA15516-qR: | Reverse | CGTAAGCTCCTCAGGCAAGT |
| RhHB1-qF： | Forward | GCATTGATGGCGAATCGGAC |
| RhHB1-qR: | Reverse | TCGGTTCCTCCTCAAGTCCA |
| RhUBI1-qF： | Forward | GGGCAATCATCTGGAATTGCTCGT |
| RhUBI1-qR： | Reverse | GCCCCCAAAGAGAAACCCTGCG |
